# Epidemiological Analysis of Obesity-related co-morbidities and mortality among post- menopausal women diagnosed with endometrial cancer: A Randomized Controlled Trial

**DOI:** 10.21203/rs.3.rs-6214415/v1

**Published:** 2025-04-08

**Authors:** Cynthia A. Thomson, Karen Basen-Engquist, Rogelio Robles-Morales, Denise J. Roe, Jennifer Erdrich, Britton Trabert, Nazmus Saquib, Michele L Cote, Lihong Qi, Dorothy S. Lane, Tracy E Crane

**Affiliations:** University of Arizona; MD Anderson Cancer Center; University of Arizona; University of Arizona; University of Arizona; University of Utah, University of Utah; Sulaiman Al Rajhi University; Indiana University Bloomington; University of California Davis; Stony Brook University; University of Miami and Sylvester Cancer Center

**Keywords:** Obesity, Endometrial Cancer, Women’s Health Initiative

## Abstract

**Objective:**

Obesity is a risk factor for endometrial cancer (EC). Five-year survival is over 80%; however, mortality rates have increased in recent years. Post-menopausal women with EC frequently present with obesity-related comorbidities or develop comorbidities after diagnosis. Survival may vary related to this comorbidity burden. This analysis describes modifiable comorbidities (diabetes, cardiovascular disease, hypertension, and fractures) among post-menopausal EC survivors and evaluates the relationship between obesity-related comorbidities and mortality after an endometrial cancer diagnosis.

**Methods:**

Epidemiological analysis of overall mortality in relation to incidence of obesity-related comorbidity (obesity, diabetes, cardiovascular disease, hypertension and fractures) after EC diagnosis. The participants are Post-menopausal women from 40 clinical sites across the U.S. participating in the Women’s Health Initiative (WHI) observational or clinical trials and diagnosed with incident EC during follow-up. Adjusted Cox proportional hazards regression models for incident EC were used to evaluate the relationship between comorbidities and all-cause mortality.

**Results:**

1661 incident cases of EC were identified. Mortality rates were 55.5% for women with EC. Prevalence increased from baseline to 18 y follow-up for each comorbidity. Regression analyses for incident EC indicated that severe obesity HR (95% CI): 2.13 (1.52, 2.97), cardiovascular disease, 1.50 (1.26, 1.78), and fracture, 1.17 (1.07, 1.27) were associated with greater overall mortality.

**Conclusions:**

Obesity-associated comorbidities are common and are associated with greater mortality in postmenopausal women diagnosed with endometrial cancer. Interventions to modify risk for comorbidity in EC survivors may improve survival and should be evaluated.

**Clinical Trial Registration:**

NCT 00000611

## 1. Introduction

Endometrial cancer (EC) is the most common gynecological cancer diagnosed in women in the U.S.; an estimated 86% of new cases occur in post-menopausal women with a mean age of onset of 60 y (1). Rates have increased steadily by 0.6% annually since 2010 in the U.S. (2), corresponding to a parallel increase in obesity rates. Risk estimates suggest that obesity is associated with an overall 85% greater risk for endometrial cancer and even greater risk in women with severe obesity (3–5). Survival after a diagnosis is considered good, with an estimated 83.6% of women still alive 5 years after their diagnosis (2). Concerning are data showing that age-adjusted mortality for endometrial cancer has increased by 1.7% annually since 2010, more than double the rise in incidence (2). Among the modifiable risk factors that have been postulated in relation to mortality after an endometrial cancer diagnosis are obesity-associated comorbidities.

Mechanistically, obesity and obesity-related comorbidities influence several of the hallmarks of cancer, including tumor cell proliferative signaling, immune modulation, evasion of growth suppression, activation of invasion, enhancing genomic instability, and deregulating cell energetics (6). Adipose serves as a source of unopposed estrogen in post-menopausal women, a mutagen that may drive DNA damage in cancer (7). Mesenchymal stem cells originating from adipose tissue can support tumor growth and proliferation, and visceral fat, common in obesity-related comorbid disease, is a source of adipokines promoting inflammation and insulin resistance (8), all of which may alter survival.

Literature substantiating the relationship between obesity and obesity-related comorbidities and EC survival are evolving. A 2004 report evaluating EC survival among 415 women suggested that a concurrent diagnosis of diabetes was associated with a 2.79 (95% confidence interval; 1.63–4.78) higher risk for overall mortality (9). Similar results of 490 EC cases in Utah showed a diagnosis of hypertension and diabetes in EC patients was associated with a 70% higher risk of death over 4.5 y of follow-up (10). Analysis of from Australia, and separately Norway, showed that comorbidities at the time of diagnosis, including obesity and diabetes, were associated with a 2–3-fold greater all-cause mortality (11, 12). A single center analysis of 2519 women with EC applying the Adult Comorbidity Evaluation (ACE)-27 (13) suggested a more than double risk of mortality or EC recurrence in women with an elevated comorbidity score (14).

While these earlier analyses were suggestive in terms of comorbidities driving endometrial cancer survival, replication in a larger U.S. sample, particularly for older women who experience a greater burden of comorbid disease, is needed. The Women’s Health Initiative (WHI), the largest study of postmenopausal women ever undertaken in the U.S., provides fertile ground for expanding knowledge in this area. Specifically, this analysis aims to describe the prevalence and incidence of modifiable obesity-related comorbidities among post-menopausal women diagnosed with endometrial cancer and to examine the association between obesity-related comorbidity and mortality after EC. Our overall goal is to determine if comorbidities which are potentially modifiable through lifestyle behavior change, are associated with survival after an endometrial cancer diagnosis in postmenopausal women.

## 2. Methods

### 2.1. Study Population

The sample was drawn from the Women’s Health Initiative (WHI), a large prospective study comprised of three overlapping randomized, controlled clinical trials (Hormone Therapy, Diet Modification, Calcium and Vitamin D) and an observational study. Details of the WHI study population have been previously described (15). Briefly, postmenopausal women between the ages of 50 and 79 years of age were recruited across 40 clinical sites across the U.S. At enrollment, eligible women were free of cancer, although a history of cancer no longer under treatment was acceptable, with a life expectancy of greater than 3 years. Enrollment occurred between 1993–1998 and included 161,808 women. All enrolled participants provided written informed consent under Human Subjects’ Committee Internal Review Board (IRB) approvals at each site and as embodied by the Declaration of Helsinki. From the WHI, our analytical sample included all women who self-reported prevalent EC at baseline as well as women who were diagnosed with incident EC after study baseline through February 28, 2021 (16). Incident EC was defined as having an adjudicated case of EC after enrollment in WHI. Women without complete data for the outcome variables or independent variables, and those who were diagnosed with EC only at the time of death, were excluded. A total of 1853 incident cases of EC were identified.

### 2.2. Data Sources

#### 2.2.1. Eligibility assessment

WHI women, including both the clinical trial participants and observational study participants (16) who self-reported EC during biannual follow-up mailed questionnaire were included in this analysis. All reported incident endometrial cancer cases were adjudicated using medical record review of clinical and pathology records by trained physician adjudicators as previously reported (16).

#### 2.2.2. Outcome assessments

The primary outcome of interest was all-cause mortality. Mortality and changes in health status were captured every six months via mailed questionnaires with follow-up telephone contact for nonresponders. WHI women reported health status on the standardized, protocol-specified questionnaire. Surrogates identified by the WHI participant at baseline provided follow-up data in the case of women who were unable or unwilling to complete the form or had expired during the observational period of query. Death was verified by medical record review and death ascertainment was complimented with annual review of the National Death Index (11) to update mortality data and cause of death.

An exploratory outcome was incident obesity-related cancers after EC diagnosis. Obesity-related cancers after EC in incident cases included: esophageal, breast, colorectal, gallbladder, kidney, liver, ovary, pancreas, thyroid, meningioma, and multiple myeloma.

#### 2.2.3. Comorbid conditions

The modifiable/treatable comorbidities of interest included obesity (defined by body mass index (BMI) category), Type 2 diabetes, CVD (angina, coronary artery bypass, carotid artery occlusion, congestive heart failure, myocardial infarction, peripheral vascular disease, percutaneous transluminal coronary angioplasty, and stroke) hypertension, and the number of incident fractures. New onset CVD and hypertension were verified through review of medical records, CVD and fracture events/diagnoses were adjudicated by trained physician adjudicators throughout the study (16). Incident diabetes was self-reported and verified through medication inventory supporting prescribed medications for the treatment of diabetes, with prior review demonstrating high validity (17).

These comorbidities were modeled as time varying, except for BMI which was fixed as measured at WHI baseline clinic visit. This means that the dataset included data for each period in time in which the woman was diagnosed with an additional comorbidity or accumulated an additional fracture, and that once a comorbidity was diagnosed it persisted through the end of follow-up.

#### 2.2.4. Covariates

Additional independent variables included suspected confounders: stage at EC diagnosis, smoking status (never, former, current), energy intake (Kcals), hormone therapy (HT) use (never, former, current), aspirin or non-steroidal anti-inflammatory drug (NSAID) use at baseline, alcohol consumption, physical activity (minutes per week), race and ethnicity, income, formal education, and parity.

### 2.3. Statistical Analyses

Descriptive statistics were calculated for variables of interest, including participant characteristics, and prevalent and incident comorbidities as well as for the exploratory evaluation of incident obesity-related cancer after EC diagnosis. To evaluate the association between mortality and modifiable comorbidities, Cox proportional hazards regressions were applied, and models were fit separately for women who had incident EC. The time origin was the age at EC diagnosis. We first fit an unadjusted model that included just the modifiable comorbidities as predictors. We then fit a fully adjusted model (covariates/confounders described above). We adjusted for WHI trial arm and treatment assignment since participants who were eligible to be randomized into the clinical trials were generally healthier than those in the observational study, and because the treatments received in the clinical trials (HT, dietary modification, and calcium + vitamin D supplementation) may have affected the outcomes of interest. For the primary analyses, we considered a p < a type I error rate of 0.05 as statistically significant. All analyses were conducted using SAS 9.4 and STATA 18 (18).

## 3. Results

A total of 1661 had EC diagnosed after study enrollment. Table 1 provides the sample demographic and clinical characteristics. The mean age of incident endometrial cancer diagnosis was 72.2 years, while age at death, among those who died during follow-up, averaged 81.2 years. Most EC cases were non-Hispanic White, with vocational or some college education, non-smokers with minimal alcohol intake and most (78.7%) were diagnosed with Stage 2 disease.

### 3.1. Incident Co-morbidities in EC Survivors

Prevalence of obesity-related comorbidities increased from baseline to 18 y follow-up for all comorbidities evaluated. This included an estimated 30% increase in hypertension (40.4% → 69.1%), 12% increase in cardiovascular disease (26.7% → 38.9%), and a doubling in incidence of diabetes (9.7% → 18.6%). Overweight and obesity was the most common comorbidity; on average, an estimated one-third of the sample had normal BMI (Table 2). Overall, hypertension was common; 40.4% of women had prevalent hypertension, and an additional 28.7% developed hypertension during follow-up (after baseline enrollment in WHI). CVD was prevalent for 26.7% of women and 12.2% of women experienced at least one incident CVD event. Diabetes was the least common comorbidity with 9.7% of women with prevalent diabetes and 8.9% with incident diabetes during a mean 17.8 y of follow-up. Incident fractures were common; 21.9% of women experienced one fracture, 7.8% had two fractures, and 3.6% had three or more fractures during follow-up.

The comorbidities evaluated focused on obesity-related comorbidities, and as such, frequently demonstrated overlap in occurrence in this sample of women where above normal BMIs were common. Further, hypertension was common and thus demonstrated higher overlap with other comorbidities, notably diabetes, obesity-related cancer, cardiovascular disease, and fractures. (Supplemental Fig. 1).

### 3.2. All-cause Mortality

During follow-up, 717 (43.2%) women with incident EC died of any cause. In adjusted models (Table 3), severe obesity, CVD, and fracture all each were associated with greater mortality with risks elevated by 2.1-fold for severe obesity, 50% for CVD and 17% for fracture, among women with EC. Adjusting for covariates showed little attenuation of unadjusted HRs (Table 3). Evaluating these relationships as a function of age (Supplemental Fig. 2) illustrates increasing HRs for all-cause mortality, across comorbidities, with advancing age.

### 3.3. Incident Obesity-related Cancers

The rate of incident obesity-related cancer was 8.97% among EC survivors (Table 4). The most common obesity-related cancer in our sample was post-menopausal breast cancer (5.42%) followed by colorectal cancer (1.63%). The mean time to diagnosis for these additional obesity-related cancers was 14.1y from EC diagnosis (76.9 y) (data not shown). The rates of new obesity-related cancers after EC were low, thus precluding robust evaluation of multivariable risk, specifically association with the presence or absence of comorbidity.

## 4. Discussion

### 4.1. Summary of Main Results

The key finding from this analysis was that obesity-related comorbidity is a significant driver of mortality after an EC diagnosis in postmenopausal women. Despite high short-term survival, severe obesity was the strongest driver of overall mortality risk among women with EC, more than doubling risk over women with normal BMI.

### 4.2. Results in the Context of Published Literature

Within our sample, obesity-related comorbidities known to drive EC risk were common. Over 34% of EC survivors were obese, over 26% had prevalent cardiovascular disease, an estimated 18.58% had diabetes, and 40% had a diagnosis of hypertension. These rates are below what is reported for obesity (43.3% - National Health Statistics data (26)) and CVD (78.2%) (27), yet similar to rates of diabetes (16–20%) reported nationally (https://gis.cdc.gov/grasp/diabetes/diabetesatlas-surveillance.html). The rates of prevalent and incident hypertension were estimated at 69%, higher than national population estimates of 39.7% (26); and also higher than the Utah EC cohort which reported estimated rates of 47% (10). The variance from national averages likely reflects the higher education, lower poverty rates, and greater access to healthcare and related screening among WHI women.

The results showed that CVD was associated with a 50% greater adjusted overall mortality risk in women with EC. An earlier report from WHI suggested there was no greater risk for CVD events following an EC diagnosis versus being free of cancer (19). Our results inform the results from Ward et al (20) in an evaluation of SEER invasive EC cases (N = 33,232) wherein CVD was the leading cause of death after EC.

Diabetes and hypertension were not associated with mortality after EC. Yet, a study in of 337 EC cases in Norway showed diabetes was associated with a 214% greater all-cause mortality (12). In the sample, diabetes was not significantly associated with cancer-specific mortality, contrary to results from Nagle and Folsom showing greater than 2-fold elevation in risk of cancer-specific mortality in EC survivors with a concurrent diabetes (9, 11). Only one earlier analysis specifically evaluated hypertension as a risk factor for mortality in EC patients. In this study of 490 patients, hypertension was associated with a 66% higher risk for overall mortality(10). Hypertension has been linked to malignant transformation of endometrial polyps; a condition identified in approximately 30% of women (21).

Fracture was associated with an elevated risk for all-cause mortality in our sample, a finding not previously reported in the literature. Fractures in older adults carry high morbidity and mortality generally (15). Additionally, fracture in older women is commonly associated with lower physical activity levels and reductions in physical activity with age are associated with elevated overall mortality (22). Physical inactivity may partially explain the associations found.

Obesity’s role in carcinogenesis is well known by the scientific community, but not well conveyed to EC patients. Ekelund et al. showed that over one-third of EC patients reported a complete unawareness of the association between obesity and EC (23). Endometrial cancer survivors’ beliefs on the benefits of exercise are promising. Lukowski et al. surveyed 106 cancer survivors and found most of believed that exercise is very or extremely likely to make them feel better physically and emotionally, maintain weight, improve sleep, and reduce the risk of chronic diseases except for cancer (24). These results imply the potential benefit of providing post-EC lifestyle interventions to this at-risk group.

Novel to this work were findings that 8.97% of EC survivors developed at least one additional obesity-related cancer after EC. Literature suggests that rates of second primary cancers vary widely among cancer patients overall (2–17%) (25), thus 9% in our sample is an appreciable amount.

### 4.3. Strengths and Weaknesses

Strengths of these analyses include the use of a rich dataset with a large sample of women who provided over 25 y of follow-up data. Our regression approach adjusted for numerous confounders and these analyses interrogate the largest US sample reported to date. Limitations exist including defining EC to include all histopathological case types given the limited information regarding EC sub-type classifications. WHI is a highly educated sample with higher-than-average access to care which may suggest our findings have greater application to EC survivors being regularly monitored within the healthcare system. Our sample was underpowered to rigorously evaluate cancer-specific mortality and we could only identify incident breast, colorectal, liver, ovary or thyroid as obesity-related cancers at baseline. Finally, we were unable to confirm the clinical effectiveness of treatment for the various comorbidities, such as blood pressure and blood glucose control that likely influenced cancer risk and mortality as well as other causes of death.

### 4.4. Implications for Practice and Future Research

These data provide support for rigorous evaluation of the impact of lifestyle behavior, and specifically weight loss interventions, to improve mortality after a diagnosis of EC among postmenopausal women.

## 5. Conclusion

In summary, obesity-related comorbidities are common after an EC diagnosis, frequently women have a multiplicity of these morbidities and interventions to address obesity are likely to reduce individual morbidities while reducing mortality in EC survivors. Given the national rise in mortality among EC survivors over the past decade, these data provide important targets for clinical intervention to potentially improve survival longer-term.

## Figures and Tables

**Table 1:** Sample characteristics of incident endometrial cancer cases in the Women’s Health Initiative (N = 1,661)

Characteristics	Mean (SD)
Age at baseline	62.7 (6.9)
Age at last observation	82.1 (6.9)
Age at death for those who died	81.2 (8.1)
Age at last observation among those surviving	82.8 (5.7)
Age at diagnosis	72.2 (7.8)
Total daily energy intake, Kcal	1722.3 (667.7)
Physical activity, minutes per week	187.2 (182.1)
Race & Ethnicity	N %
Non-Hispanic White	1,505 (90.6%)
Black	83 (5.0%)
Hispanic	23 (1.4%)
American Indian or Alaskan Native	23 (1.4%)
Asian or Pacific Islander	2 (0.1%)
Other Race	20 (1.2%)
Not Reported	5 (0.3%)
Formal Education	
Less than High School	44 (2.6%)
High School or GED completed	239 (14.4%)
Vocational training, technical or some college	537 (32.3%)
College Degree	207 (12.5%)
Some post-graduate or professional school	245 (14.8%)
Graduate Degree	378 (22.8%)
Missing	11 (0.7%)
Income	
Less than $20,000	188 (11.3%)
$20,000 to $34,999	332 (20.0%)
$35,000 to $49,999	311 (18.7%)
$50,000 to $74,999	351 (21.1%)
$75,000 and greater	358 (21.6%)
Missing or Unknown	121 (7.3%)
Smoking Status at Baseline	
Never Smoked	881 (53.0%)
Formerly Smoked	702 (42.3%)
Currently Smoke	78 (4.7%)
Alcohol Intake	
Does not drink alcohol	139 (8.4%)
Drank alcohol in the past	258 (15.5%)
Minimal (< 1 drink per week)	597 (35.9%)
Moderate (1–<7 drinks per week)	440 (26.5%)
Heavy (7 + drinks per week)	227 (13.7%)
Parity	
Never pregnant	205 (12.3%)
Never had term pregnancy	56 (3.4%)
1	157 (9.5%)
2	428 (25.8%)
3	420 (25.3%)
4	215 (12.9%)
5+	174 (10.5%)
Missing	6 (0.4%)
Menopausal Hormone Therapy	
Never	767 (46.2%)
Former	236 (14.2%)
Current	656 (39.5%)
NSAID Use	
Using NSAID’s	329 (19.8%)
Not using NSAID’s	1,332 (80.2%)
SEER Stage (in incident EC)	
1	15 (0.9%)
2	1,308 (78.7%)
3	238 (14.3%)
4	81 (4.9%)
Unknown Stage	19 (1.1%)

**Table 2:** Obesity-related comorbidities^a^ among post-menopausal women participating in the Women’s health Initiative and diagnosed with endometrial cancer (N = 1,661)

Variable	n	%
BMI category at baseline		
Underweight (< 18.5)	14	0.73
Normal (18.5–24.9)	605	31.76
Overweight (25.0–29.9)	628	32.97
Obesity I (30.0–34.9)	371	19.48
Obesity II (35.0–39.9)	164	8.61
Severe obesity III (≥ 40)	123	6.46
Diabetes: Prevalent	185	9.71
Diabetes: Incident	169	8.87
Cardiovascular disease (CVD)^d^: Prevalent	508	26.67
CVD: one incident event	233	12.23
CVD: two incident events	49	2.57
CVD: three incident events	8	0.42
Hypertension: Prevalent	769	40.37
Hypertension: Incident	546	28.66
Incident fractures		
One fracture	418	21.94
Two fractures	150	7.87
Three or more fractures	69	3.61

a Incident comorbidities developed after EC diagnosis.

b Endometrial cancer reported on medical history at time of enrollment in the Women’s Health Initiative

c Endometrial cancer diagnosed and adjudicated during participation in the Women’s Health Initiative

d CVD defined as: angina, coronary artery bypass, carotid artery occlusion, congestive heart failure, myocardial infarction, peripheral vascular disease, percutaneous transluminal coronary angioplasty, and stroke

**Table 3:** Hazard ratios for all-cause mortality, unadjusted and adjusted, in WHI women diagnosed with endometrial cancer (EC)

	Unadjusted		Adjusted	
Comorbidities	HR	95% CI	HR	95% CI
Incident EC				
BMI Category at Baseline				
Underweight (< 18.5)	0.76	0.24, 2.40	0.68	0.21, 2.18
Overweight (25.0–29.9)	1.12	0.91, 1.37	1.10	0.89, 1.36
Obesity I (30.0–34.9)	1.18	0.94, 1.48	1.19	0.94, 1.51
Obesity II (35.0–39.9)	1.57	1.22, 2.03	1.62	1.22, 2.15
Severe obesity III (> = 40)	1.91	1.42, 2.57	2.13	1.52, 2.97
Diabetes	1.33	1.10, 1.60	1.19	0.97, 1.46
Cardiovascular disease	1.45	1.23, 1.71	1.50	1.26, 1.78
Hypertension	0.95	0.80, 1.13	0.95	0.80, 1.14
Fracture count	1.19	1.09, 1.29	1.17	1.07, 1.27

Cox proportional hazards regression models were fit for the incident endometrial cancer cases. Reference groups are: BMI Normal (18.5–24.9), absence of CVD, absence of diabetes, absence of hypertension, no incident fracture. Diabetes, cardiovascular disease, hypertension, and the number of incident fractures in the model are time-varying. Covariates for adjusted models were study arm, education, income, race/ethnicity, current smoker, minutes per week of exercise, total energy (Kcal) consumed, alcohol intake, current HRT use at baseline, former HRT use at baseline, NSAID or aspirin use at baseline, metformin use at baseline, and parity.

**Table 4:** Incident Obesity-related cancers among women who diagnosed with endometrial cancer (EC)

Variable			n	%
Obesity-related cancers: Incident	200	10.50	149	8.97
Breast cancer	116	6.09	90	5.42
Colorectal cancer	60	3.15	27	1.63
Gallbladder cancer	0	0.00	1	0.06
Kidney cancer	8	0.42	9	0.54
Liver cancer	3	0.16	3	0.18
Ovarian cancer	8	0.42	25	1.51
Pancreatic cancer	0	0.00	8	0.48
Thyroid cancer	5	0.26	2	0.12
Meninges cancer	0	0.00	0	0.00
Multiple myeloma	4	0.21	1	0.06
Esophageal cancer	2	0.10	3	0.18

## Data Availability

https://www.whi.org/md/working-with-whi-data

## Abbreviations

EC: Endometrial Cancer
WHI: Women’s Health Initiative
ACE: Adult Comorbidity Evaluation
BMI: Body Mass Index
CVD: Cardio Vascular Disease
Kcal: Kilocalories
HT: Hormone Therapy
NSAID: Non-Steroidal Anti-Inflammatory Drug
